# Simultaneous Measurement of Temperature and Pressure Based on Fabry-Perot Interferometry for Marine Monitoring

**DOI:** 10.3390/s22134979

**Published:** 2022-07-01

**Authors:** Shengqi Zhang, Yongchang Mei, Titi Xia, Zihan Cao, Zhengyong Liu, Zhaohui Li

**Affiliations:** 1 Guangdong Provincial Key Laboratory of Optoelectronic Information Processing Chips and Systems, School of Electronics and Information Technology, Sun Yat-sen University, Guangzhou 510275, China; zhangshq58@mail2.sysu.edu.cn (S.Z.); meiych3@mail2.sysu.edu.cn (Y.M.); xiatt5@mail2.sysu.edu.cn (T.X.); caozh7@mail2.sysu.edu.cn (Z.C.); 2 Southern Marine Science and Engineering Guangdong Laboratory (Zhuhai), Zhuhai 519082, China

**Keywords:** fiber-optic sensor, Fabry-Perot interferometer, FBG, temperature and pressure sensing, marine monitoring

## Abstract

The temperature and pressure of seawater are of great importance to investigate the environmental evolution for the research of ocean science. With this regard, we proposed and experimentally demonstrated a seawater temperature and pressure sensor realized by a polyimide (PI) tube-based Fabry-Perot interferometer (FPI) together with a fiber Bragg grating (FBG). Benefiting from the higher thermo-optical coefficient and larger elasticity of polymer than the fused silica fiber, the sensitivity of the sensor is largely improved. The FBG is used to compensate the cross effect of the temperature. The measured temperature and pressure sensitivities of the sensor are 18.910 nm/°C and −35.605 nm/MPa, respectively. Furthermore, the temperature and pressure information measured by the sensor can be achieved simultaneously using the sensitivity matrix method. In addition, the proposed sensor has advantages of easy fabrication, compact size, as well as capability of multiplexing and long-distance measurement, making it competitive and promising during the marine monitoring.

## 1. Introduction

The temperature and depth of seawater are important fundamental parameters in oceanology and are crucial for building an accurate distribution of the seawater temperature. It is essential to monitor the marine information accurately for the marine hydrological survey and the exploitation of marine resource, marine construction operations, marine fisheries, and other marine activities. 

At present, most of marine environmental monitoring devices are electrical equipment such as conductivity-temperature-depth (CTD), which is generally expensive, complex, bulky and difficult to deploy, prone to electromagnetic interference, and cannot meet the requirements of extreme environmental monitoring. In addition, each environmental parameter is monitored by discrete equipment. To achieve large-scale monitoring in a marine, a large number of sensor arrays are needed. In contrast, optical fiber temperature/pressure sensors show prominent advantages of small size, compact structure, corrosion resistance, electromagnetic immunity, as well as capability of multiple parameters sensing, multiplexing and long-distance measurement. It is also easy to integrate with optical communication system. In addition to the conventional single mode fiber (SMF) designed for the optical communication, various specialty optical fibers have been demonstrated as well. Those fibers employ novel structure and materials to achieve microstructured fibers [1] or multimaterial fibers [2,3,4], providing more opportunities and feasibility to develop high performance sensors.

Over the last couple of years, various types of optical fiber pressure and temperature sensors have been extensively investigated and developed. Most of them are on the basis of Mach–Zehnder interferometer (MZI) [5,6,7], fiber Bragg gratings (FBG) [8,9,10], Sagnac interferometer (SI) [11,12], and the Fabry-Pérot interferometer (FPI) [13,14,15,16,17,18,19,20], etc. Among these sensing structures, the FPI-based sensors have attracted increasing attention due to simple structure, compact size and high accuracy. In an attempt to measure the pressure and temperature simultaneously, several approaches have been proposed and utilized to manufacture the FPI sensors. For instance, in 2002, Qi et al. presented a fiber-optic pressure and temperature sensors consisting of two optical fibers and a silica capillary tube. The air gap can change with the external pressure or temperature. Experimental results show that the self-calibrating interferometric/intensity-based (SCIIB) system achieves an accuracy of 0.1% in a working range of 0–8000 psi for the pressure sensing and the measured temperature can be up to 600 °C. The results of long-term test conducted in the oil field show high potential applications in down-hole monitoring [13]. In 2019, Li et al. proposed a temperature and pressure sensor based on FBG cascaded with a FP cavity. The sensor can withstand at 100 MPa with a repeatability of 0.01%, and has been demonstrated in the test of the Shengli Oil Field [14]. However, the sensitivity is not high as the pure silica material is utilized.

In addition to being employed in oil field, the FPI-based sensors for pressure and temperature measurement were also applied in the underwater monitoring. In 2014, Duraibabu et al. presented an ocean temperature and pressure sensor combined extrinsic FPI (EFPI) sensor and femtosecond laser inscribed FBG. A single mode fiber (SMF) and a polished 10µm multimode fiber (MMF) were spliced to the glass capillary tube. The MMF forms the EFPI diaphragm [15]. In the year of 2017, Duraibabu et al. further refined the sensor structure and used it for practical pressure and temperature measurements in fresh water and in the ocean [16]. Similarly, in 2016, Bai et al. proposed a temperature and pressure optical fiber sensor based on FPI. The sensor included an incident SMF, a capillary pure silica tube, and the gap was filled with refractive index matching liquid. A temperature sensitivity of 6.95 nm/°C was achieved and the structure showed a pressure sensitivity of 0.15 nm/Pa [17]. However, the usage of refractive index matching liquid made it not reliable in actual application.

Apart from the FBGs and capillary pure silica tubes, various polymers have also been investigated to fabricate FPI sensors for the pressure and temperature measurement. In 2015, Sun et al. investigated a polymer-capped FPI for simultaneous measurement of temperature and pressure, which is based on a polymer capped on the end face of a SMF. The temperature sensitivity and pressure sensitivity of the proposed sensor were 249 pm/°C and 1130 pm/MPa, respectively [18].

In addition, specialty optical fibers have been utilized to sense the temperature and pressure based on FPI. In 2021, Ma et al. proposed a structure of sapphire-derived fiber together with a silica capillary-based compound FPI to realize a highly sensitive pressure and temperature sensor. In this approach, the demonstrated pressure sensitivity was 5.19 nm/MPa; even the temperature was up to 700 °C, showing a temperature sensitivity of 0.013 nm/°C [19]. In this work, the cavity was made of pure silica capillary, leading to relatively low sensitivity. The temperature compensation was realized via another FP cavity consisting of sapphire-derived fiber, which increased the complexity of the structure. In the same year, Zhao et al. proposed a sensing structure of a ring shape using polymer coated no-core fiber (NCF) to measure the pressure and temperature. Based on such structure, there was anti-resonance occurring in NCF, and the resonance dip showed a nearly linear response to the change of temperature and displacement. With a proper compensation algorithm, this sensing structure could give a temperature sensitivity of −5.098 nm/°C and pressure sensitivity of −2.368 nm/MPa, respectively [20]. However, since the fiber ring structure was employed, it made the system sophisticated in the package, especially aiming for the marine monitoring.

In this paper, we proposed and experimentally demonstrated a seawater temperature and pressure sensor realized with polyimide (PI) tube-based FPI and FBG. The end faces of two SMFs were glued to a PI tube with UV glue to form the FP cavity. An FBG was designed adjacent to the FP cavity measuring the temperature so as to compensate for the temperature effect induced on the PI tube-based FP cavity. Compared with other sensors, the PI tube-based FPI sensor can achieve a higher sensitivity owing to the high thermo constant and large elasticity of the polymer. The experiments show that the maximum temperature and pressure sensitivities of the proposed sensing structure could be 18.910 nm/°C and −35.605 nm/MPa, respectively. In addition, this sensor is a potential candidate to measure the parameters of the seawater due to the advantages of simple fabrication, compact size and high sensitivity.

## 2. Sensing Principle and Fabrication

**Figure 1:**
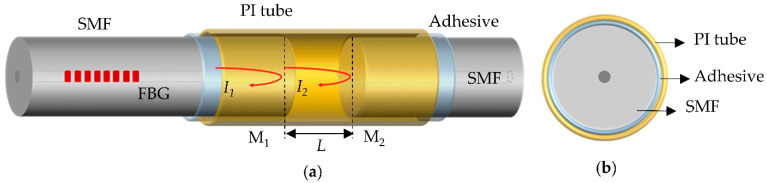
(**a**) Schematic diagram of the proposed pressure sensor based on the structure of FPI and FBG. (**b**) Cross-sectional view of the PI tube end.

The schematic structure of the temperature and pressure sensor realized with PI tube- based FPI is illustrated in Figure 1. The ends of the fiber with the FBG and a SMF are inserted into the PI tube (purchased from D.SOAR Ltd., Shenzhen, China), and the two fibers were fixed with UV glue (DSM 950-200) at two ends of the tube. The two ends without coating were introduced in the FPI, labeled as reflection mirror M_1_ and M_2_, which formed the FP cavity with a cavity length of *L*. Based on Fresnel formula, the reflectivity of mirror M_1_ and M_2_ are 3.5% approximately. The FBG with a Bragg wavelength of ~1552.8 nm was inscribed in a SMF by UV laser via phase-mask technique. The inner and outer diameters of the PI tube were 130 µm and 160 µm, respectively. An optical interrogator (Micro Optics, si155 HYPERION) with a wide wavelength range covering from 1460–1620 nm was utilized to measure the interference spectrum of the sensor. It has a resolution of ~1 pm and the sampling frequency is 10 Hz, allowing for a precise measurement of the spectral change.

In principle, the incident light coming out from the interrogator and propagating through the sensor is reflected at the M_1_ and M_2_ interface. The two reflected beams from the two reflection mirrors are combined into the SMF, causing a backward travelling interference signal acquired by the interrogator.

Particularly for the FPI sensing structure demonstrated in this work, the final intensity of the reflection light can be described as

(1) $$I_{r}=I_{1}+I_{2}+2\sqrt{I_{1}I_{2}}\cos\left(\varphi \right),$$

where $I_{1}$ and $I_{2}$ are light intensities of the two reflected beams that propagate in different paths. φ is the phase difference between the two beams, which is formulated as

(2) $$\varphi =\frac{4\pi nL}{\lambda },\;$$

where *n* is the refractive index of the medium in the cavity, which equals to 1 since the medium inside the cavity is air (i.e., *n* ≈ 1); *λ* is the optical wavelength in the medium. The two reflected beams interfere with each other constructively or destructively, depending on the phase difference of the two beams, namely the length of FP cavity.

Regarding the FP cavity, changes in the external temperature (∆*T*) and pressure (∆*P*) alter the cavity length (∆*L*), resulting in a corresponding spectral shift. When the external pressure changes, the induced length variation of the PI tube-based FP cavity can be described by [13]

(3) $$\Delta L=\frac{L_{0}\cdot d_{o}^{2}}{E_{PI}\left(d_{o}^{2}-d_{i}^{2}\right)}\cdot \left(1-2\mu \right)\cdot \Delta P,$$

where $L_{0}$ is the distance between the two adhesive positions, ∆*P* represents the change of the external pressure, $d_{i}$ and $d_{o}$ are the inner and outer diameters of the PI tube, $E_{PI}$ and μ are the Young’s modulus and Poisson’s ratio of PI tube.

The adhesive UV glue is a thin-walled cylinder type sandwiched between PI and SMF, so the main pressure it receives is axial pressure. The cross-sectional area of the UV glue can be approximated as

(4) $$A=\pi \cdot \left[{\left(\frac{d_{i}}{2}\right)}^{2}-{\left(\frac{d_{SMF}}{2}\right)}^{2}\right]\approx \pi \cdot d_{i}\cdot \delta ,$$

where $d_{i}$ and $d_{SMF}$ are the inner diameter of the PI tube and diameter of SMF, δ is the thickness of the UV glue. Basically, the FP cavity length changes with the compression of the adhesive material under the pressure, and can be described as

(5) $$\Delta L=E_{UV}\cdot L_{0}\cdot \frac{\Delta P\cdot \pi \cdot {\left(\frac{d_{i}}{2}\right)}^{2}}{A}=E_{UV}\cdot L_{0}\cdot \frac{\Delta P\cdot d_{i}}{4\delta },$$

where $E_{UV}$ is the Young’s modulus of UV glue. By contrast, FP sensor with polymers (PI and UV glue) is more sensitive to pressure changes, due to its smaller Young’s modulus than fibers.

According to Equation (2), when the phase difference satisfies:

(6) $$\frac{4\pi nL}{\lambda }=\left(2m+1\right)\pi ,\;\left(m=1,2,3\cdots \right),$$

the interference dips appear in the spectrum. *m* stands for the order of the resonance dips. The corresponding wavelength $\lambda_{m}$ of the interference dip can be described as

(7) $$\lambda_{m}=\frac{4\pi nL}{\left(2m+1\right)\pi }$$

From Equation (7), the FP cavity length variation can induce the wavelength change of the interference dip. Once a pressure variation occurs, the FP cavity length will change accordingly, which eventually leads to the spectral shift. Basically, the increase of the pressure leads to a blue shift of the spectral wavelength of the interference due to the compression of the cavity length.

Additionally, the FP cavity length also changes with the external temperature because of thermal expansion of the polymers. Therefore, an FBG is cascaded with the FP cavity, and the influence of temperature on the FP cavity can be compensated.

According to the mode coupling theory, the Bragg wavelength of an FBG can be given by the expression:

(8) $$\lambda_{B}=2n_{eff}\Lambda ,$$

where Λ is the pitch of the grating and $n_{eff}$ is the effective index of refraction of the fundamental mode. The shift in Bragg wavelength caused by the strain and temperature can be expressed as follows [10]:

(9) $$\frac{\Delta \lambda }{\lambda }=\left(1-P_{e}\right)\varepsilon +\left(\alpha_{s}+\xi_{s}\right)T,$$

where $P_{e}$ is the photo-elastic coefficient, $\xi_{s}$ is the thermo-optical coefficient, $\alpha_{s}$ is the thermo-elastic coefficient, ε and *T* represent the applied strain and surrounding temperature. Because of the elasticity of polymers larger than fibers, the FBG is insensitive to pressure changes. As reported previously, the pressure response of a pure FBG inscribed in silica SMF is about ~3 pm/MPa [21], which is much smaller compared to the PI-based FPI. Therefore, a change in surrounding pressure and temperature will lead to changes of the reflected spectrum of FBG.

## 3. Experimental Verification and Results

### 3.1. Pressure Measurement

**Figure 2:**
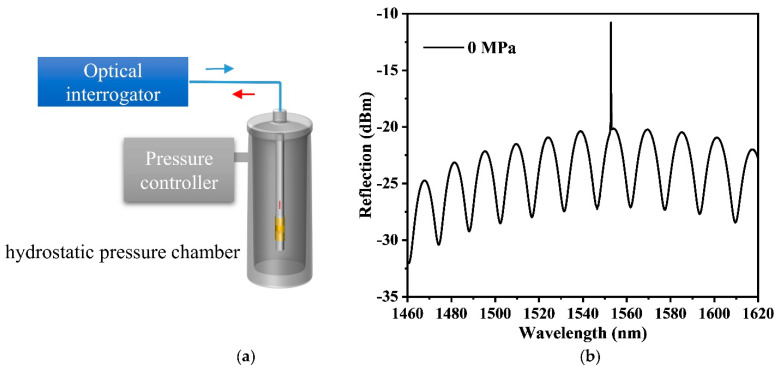
(**a**) Schematic experimental setup of the pressure measurement. (**b**) Reflected spectrum of the sensor.

To investigate the pressure sensing characteristics of the PI tube-based FPI sensor, an experimental setup was established, as exhibited in Figure 2a. During the pressure sensing experiment, the PI tube-based FP sensor was placed in a closed hydrostatic pressure chamber with controllable pressure. The actual pressure inside the chamber was calibrated by a digital pressure gauge, which is used as feedback for the controller to achieve a precise pressure environment. The optical interrogator (Micro Optics, si155 HYPERION) with a wide wavelength range covering from 1460–1620 nm was connected to the sensor to measure the interference spectrum. It has a resolution of ~1 pm and the sampling frequency is 10 Hz. Generally, the output spectrum of the sensor is recorded and the certain wavelength dip for FP cavity or wavelength peak for FBG are traced manually when the pressure changes to one stage. An example of the reflected interference spectrum at room temperature and zero pressure is plotted in Figure 2b.

**Figure 3:**
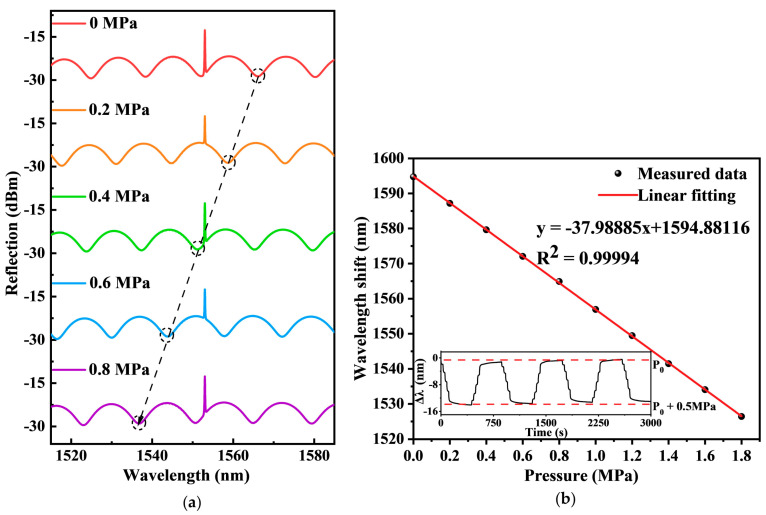
(**a**) The reflected spectrums shift with respect to the pressure applied to the sensor. (**b**) The measured wavelength shift with respect to the pressure applied to the FPI.

In the experiment, the pressure in the hydrostatic chamber was increased from 0 to 5 MPa with a step of 0.5 MPa, and the actual pressure was calibrated by a pressure gauge connected to the chamber. The corresponding interference spectra under different pressures were recorded, shown as Figure 3. As the increase of the pressure, the interference shows a corresponding blue shift, verifying the theoretical analysis. The dash line in Figure 3a represents the spectrum shift with respect to the pressure applied to the FPI from 0 to 0.8 MPa with a step of 0.2 MPa. In addition, a cycle test with increasing and decreasing pressure of 0.5 MPa was conducted, and the result is illustrated in the inset of Figure 3b. As it can be observed, the wavelength shift could recover to the original state after changing the pressure. Thus, the sensor has good response to pressure changing repeatedly.

**Figure 4:**
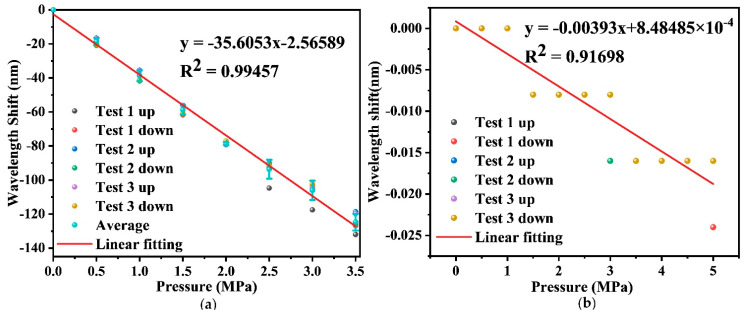
(**a**) The measured wavelength shift with respect to the pressure applied to the FPI; (**b**) the measured response of the center wavelength of FBG versus the pressure.

In an attempt to test the repeatability of the measurement, the response of dip wavelength to the pressure increasing and decreasing were measured repeatedly for three times. The experimental results of pressure sensing are displayed in Figure 4. It can be observed that the wavelength shifts show inversely proportional to the applied pressure, and the achieved pressure sensitivities of the FPI and the FBG are −35.605 nm/MPa and −0.004 nm/MPa, respectively. Since the pressure response of the FBG is quite small, as plotted in Figure 4b, it is difficult to acquire the accurate Bragg wavelength shift as the pressure changes. Therefore, the FBG can be utilized to compensate the temperature effect to some extent. Because of the results showing in Figure 4a, a relatively good repeatability of the sensor during the repeated measurements is achieved, and the average error is 3.14 nm, revealing a measurement accuracy of ~88.19 kPa. Given that the resolution of the interrogator used is ~1 pm, the resolution of the pressure measurement can be estimated as ~28 Pa although the measurement accuracy is relatively higher. It is noted that the dip wavelength of the interference spectrum was traced manually using ENLIGHT software during the measurement to ensure the accurate wavelength shift. In practical, better algorithms based on deep learning [22] are under investigation to solve the issue that dip wavelength shift exceeds one free spectral range (FSR).

### 3.2. Temperature Measurement

**Figure 5:**
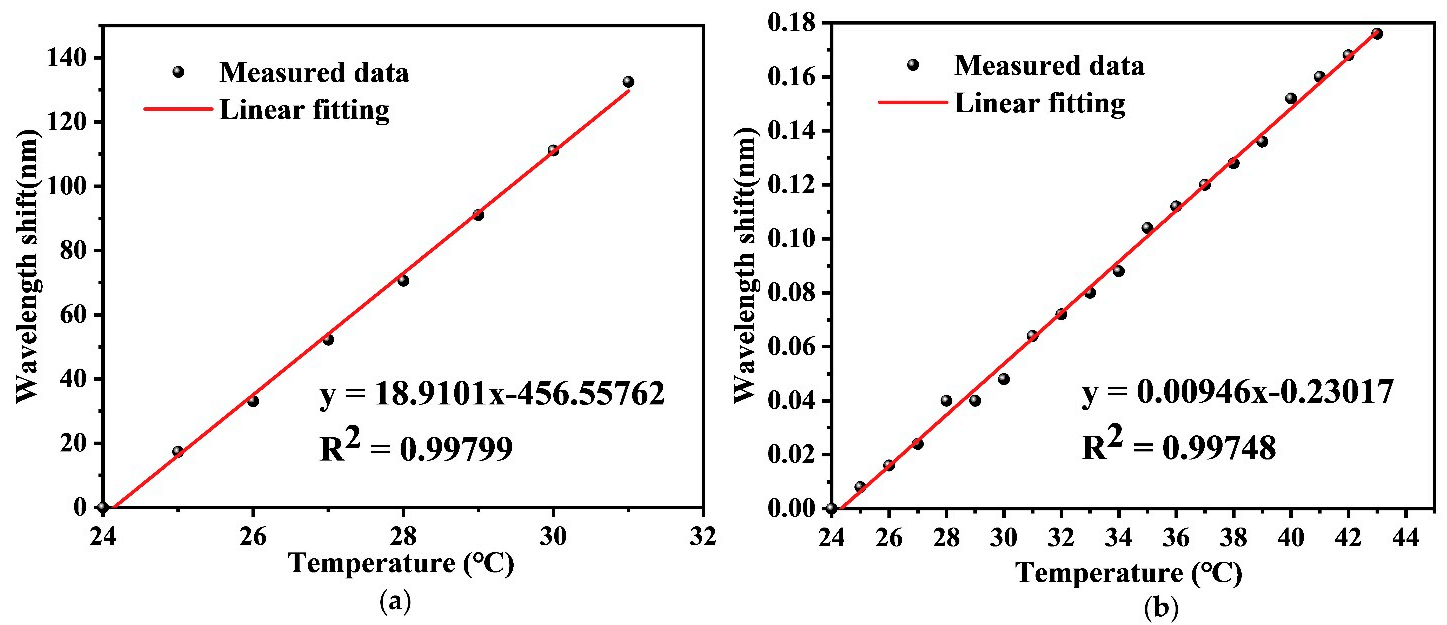
(**a**)The wavelength shift of the FPI response to temperature; (**b**) The relationship between the center wavelength of FBG and temperature.

For the experimental measurement of temperature, the sensor was placed in a heating oven. The optical interrogator (Micro Optics, si155 HYPERION) was connected with the sensor to record the spectral shifts. Given a constant pressure, the temperature was precisely controlled and increased from 24 °C to 43 °C in an increment of 1 °C. The wavelength shift of the FPI interference dip and the center wavelength shift of FBG can be recorded by the optical interrogator. As a result, the wavelength shift with respect to the applied temperature is plotted in Figure 5. It can be discovered that the dip wavelength shows a linear response to the temperature, and the measured temperature sensitivities of the FPI and the FBG obtained are 18.910 nm/°C and 9 pm/°C, respectively. It is noted that owing to the lower sensitivity to temperature, the measurement temperature of the FBG is up to 43 °C, as plotted in Figure 5b.

### 3.3. Two-Parameter Demodulation

Finally, a sensitivity matrix can be used to obtain the two measurands through the output spectral signal, because of good linearity of the proposed sensor. The total wavelength shifts of FBG peak and interference dip are induced by the temperature and pressure simultaneously. By converting the linear relationship between the wavelength shift and measurand to a matrix form, the cross-sensitivity could be deduced as

(10) $$\left[\begin{array}{c} \Delta \lambda_{1}\\ \Delta \lambda_{2} \end{array}\right]=\left[\begin{array}{cc} S_{T1} & S_{P1}\\ S_{T2} & S_{P2} \end{array}\right]\left[\begin{array}{c} \Delta T\\ \Delta P \end{array}\right]$$

where $\Delta \lambda_{1}$ and $\Delta \lambda_{2}$ represent the shifts of the FPI interference spectrum and peak wavelength of the FBG, ΔT and ΔP are the change of the applied temperature and pressure, respectively. $S_{T1}$, $S_{P1}$, $S_{T2}$ and $S_{P2}$ are the temperature and pressure sensitivities of FBG and FP cavity. Equation (10) can be deduced as the following format shown in Equation (11) to obtain the pressure and temperature. Thus, the ambient temperature and pressure change can be calculated via the cross-sensitivity matrix by substituting the measured wavelength changes of the interference spectrum and FBG.

(11) $$\left[\begin{array}{c} \Delta \lambda_{1}\\ \Delta \lambda_{2} \end{array}\right]=\left[\begin{array}{cc} 0.009\;\mathrm{nm}/{}^{\circ}C & -0.004\;\mathrm{nm}/\mathrm{MPa}\\ 18.910\;\mathrm{nm}/{}^{\circ}C & -35.605\;\mathrm{nm}/\mathrm{MPa} \end{array}\right]\left[\begin{array}{c} \Delta T\\ \Delta P \end{array}\right]$$

## 4. Discussion

Based on the experimental results, the proposed sensing structure constructed by PI tube- based FPI and FBG performs promisingly as concerns the temperature and pressure. As a comparison, Table 1 summarizes the measurement performance of various sensing structures reported previously for distinguishing the temperature and pressure simultaneously. Basically, the sensitivity shows a large difference depending on the principle and structure used. The sensitivity of some sensors consisting of all fiber are relatively low because slight variation could be induced on fiber by the pressure. In contrast to the pure silica, the sensor with polymer possesses higher sensitivity owing to its higher thermo-optical coefficient and larger elasticity of polymer than fiber. The higher sensitivity of FPI realized by MMF diaphragm can be up to 15 nm/kPa, where the diaphragm is formed by cleaving and polishing a MMF to achieve a thickness of 7–8 µm [16]. However, such interferometric configurations of MMF diaphragm are relatively complex in terms of the fabrication since precise control of the diaphragm thickness is required. Regarding the fabrication, the proposed sensing structure integrates SMF with a sensitive PI tube, making it easy to achieve mass production. Compared to the specialty optical fiber, for example the SMF for fabrics to enhance the acoustic pressure sensitivity of the fiber [1], the feature of the current sensor employs a Fabry-Perot interferometer as a sensitivity enhancement structure. As a further improvement, it is possible to replace the SMF using specialty optical fiber to increase the sensing performance of the fiber.

## 5. Conclusions

In conclusion, aiming for the real-time monitoring of the temperature and pressure under the sea, we have proposed and experimentally demonstrated a hybrid sensor realized with PI tube- based FPI and FBG to implement the simultaneous measurement of both parameters. The experimental results show that the wavelength shifts of the interference spectrum exhibit a linear response to the applied temperature and pressure. As a result, the measured temperature and pressure sensitivities based on such sensing structure are 18.910 nm/°C and −35.605 nm/MPa, respectively. Furthermore, by establishing a sensitivity matrix, the applied temperature and pressure are possible to be demodulated simultaneously. As the general principles of FPI and FBG are employed, the sensor possesses the features of easy fabrication, compact structure, as well as high sensitivity, which makes it competitive for the monitoring of temperature and pressure under the sea. By employing proper protection of the proposed sensor, it is promising for use in oceanic monitoring, especially in the application of depicting the temperature and depth profile.

## Figures and Tables

**Table 1:** Comparison of the temperature and pressure sensors based on various principles and structures.

Sensing Principle	Sensing Structure	Temperature Sensitivity (nm/°C)	Pressure Sensitivity (nm/MPa)	Structural Complexity
FBG	Aluminum diaphragm [9]	0.0178	2.43	Simple
	Thin-walled oval cylinder [10]	0.02978	1.198	Simple
MZI	PDMS-OMCI [5]	−2.283	3.301	Moderate
	DMF-MZI [2]	0.256	0.437	Simple
	PDMS-MZI [7]	−7.41	13.31	Moderate
SI	PDMS-OMCSL [12]	−2.133	3.416	Moderate
FPI	MM diaphragm-EFPI-FBG [16]	0.0125	1.5 × 10^4^	Complex
	Polymer capped-SMF [18]	0.249	1.13	Moderate
	Silica capillary-SDF [19]	0.013	5.19	Moderate
	Ring-shaped coating-SMF-NCF [20]	−5.098	−2.368	Complex
	This work	18.910	−35.605	Simple

## Data Availability

The datasets for this study are available from the corresponding author upon reasonable request.
